# Regional Grey and White Matter Changes in Heavy Male Smokers

**DOI:** 10.1371/journal.pone.0027440

**Published:** 2011-11-04

**Authors:** Rongjun Yu, Liyan Zhao, Lin Lu

**Affiliations:** 1 Department of Psychology, South China Normal University, Guangzhou, China; 2 MRC Cognition and Brain Sciences Unit, University of Cambridge, Cambridge, United Kingdom; 3 National Institute on Drug Dependence, Peking University, Beijing, China; The University of Hong Kong, Hong Kong

## Abstract

Cigarette smoking is highly prevalent in the general population but the effects of chronic smoking on brain structures are still unclear. Previous studies have found mixed results regarding regional grey matter abnormalities in smokers. To characterize both grey and white matter changes in heavy male smokers, we investigated 16 heavy smokers and 16 matched healthy controls, using both univariate voxel-based morphometry (VBM) and multivariate pattern classification analysis. Compared with controls, heavy smokers exhibited smaller grey matter volume in cerebellum, as well as larger white matter volume in putamen, anterior and middle cingulate cortex. Further, the spatial patterns of grey matter or white matter both discriminated smokers from controls in these regions as well as in other brain regions. Our findings demonstrated volume abnormalities not only in the grey matter but also in the white matter in heavy male smokers. The multivariate analysis suggests that chronic smoking may be associated with volume alternations in broader brain regions than those identified in VBM analysis. These results may better our understanding of the neurobiological consequence of smoking and inform smoking treatment.

## Introduction

Cigarette smoking has been increasingly prevalent in economically developing regions of the world. With a population of 1.3 billion, China, for example, is now the world's largest producer and consumer of tobacco and bears a large proportion of deaths attributable to smoking. There is strong evidence that tobacco smoking causes an increased risk of cancer, vascular disease, and respiratory disease in China and elsewhere [1]. Cross-sectional studies on smoking and quality of life suggest that smokers have poorer quality of life than nonsmokers [2], [3]. Despite that smoking has serious consequences on physical and mental health, how chronic smoking affects human brain structure is still unclear.

Recent structural neuroimaging studies have begun to examine the brain abnormality in smokers, focusing on regional grey matter (GM) volume or density. The first morphometry study found that GM density in dorsal lateral prefrontal cortex (DLPFC), ventral lateral prefrontal cortex (VLPFC), and right cerebellum was significantly smaller in smokers than in controls [4]. Consistent with these findings, smaller GM density or volume in lateral prefrontal cortex in smokers was also found in another two studies [5], [6]. However, the findings regarding other brain regions in smokers are mixed. For example, reduced GM density in cerebellum in smokers was reported in two studies but not in other studies [4], [7]. The findings regarding the insular GM density are contradicting. One study found smaller GM density in insula in smokers [5], but another study from the same group reported increased insula GM density [6]. A recent voxel-based morphometry (VBM) study found reduced GM density in anterior cingulate cortex [8]. By contrast, in Brody et al. (2004)'s study, smaller volumes in the left dorsal anterior cingulate cortex (ACC) was only found in hand-drawn regions of interest (ROI) analysis but not in their VBM analysis. A potential limitation of prior studies is the inclusion of smokers with variable smoking years, ranging from 1 to more than 25 years [4]–[8].

Regional white matter (WM) in smokers was not examined in these previous studies. Only one study investigated the effects of smoking on lobar GM and WM volumes in heavy drinkers [9]. Using ROI analysis, it was found that temporal lobe volume was larger in alcohol dependent smokers than in alcohol dependent non-smokers [9]. However, in that study, the alcohol drinking levels were not balanced between groups. To date, no study has investigated WM alternations in heavy smokers using VBM.

Compared to the ROI approach, which manually delineate GM/WM volumes in pre-specified regions only, VBM allows for examining the entire brain on a voxel-by-voxel basis in a fully automated manner, without having to specify in advance regions of interest. However, VBM offers high exploratory power but with moderate statistical power, as corrections for multiple comparisons are required in order to limit the occurrence of false positives. This mass-univariate approach may be too conservative to detect subtle morphological differences [10]. A multivariate whole-brain classification approach, using support vector machine (SVM), was used to detect differences in the morphology of brain regions. SVM takes into account interregional correlations and is suitable to study fine-grained neural representations even when the spatial resolution of human neuroimaging is limited [11]. Using structure neuroimaging data, SVM has successfully been used to discriminate controls from patients, including autism [10], boys with Fragile X syndrome [12], and depressive individuals [13]. The aim of the study was to examine structural brain abnormalities in smokers compared with HC using both univariate between-group comparisons (i.e. VBM) and multivariate pattern classification methods (i.e. SVM, see **Materials and Methods**).

## Results

The smoker (n = 16) and the non-smoker (n = 16) groups were selected to be matched for age (mean±SD: smokers 41.6±5.5 vs. non-smokers 39.2±4.5) and education (mean±SD: smokers 10.9±2.2 vs. non-smokers 12.2±2.5), p values >0.1. Smokers smoke 20.6±7.4 (mean±SD) cigarettes per day and the average smoke years are 21.1±3.9 (mean±SD). The mean score on the Fagerström Test for Nicotine Dependence (FTND) questionnaire was 7.19±1.42 (mean±SD), indicating heavy nicotine dependence.

### Voxel-based morphometry

**Figure 1:**
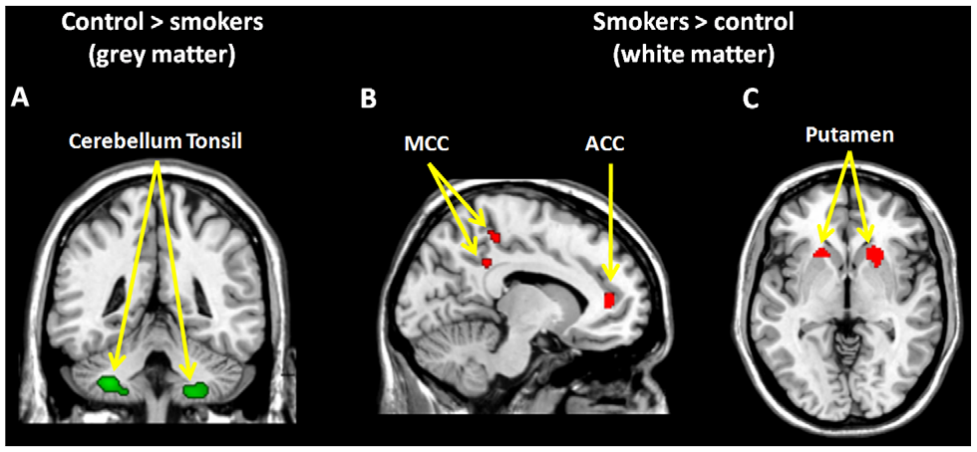
Regions that showed a significant structural difference between heavy smokers and controls from the VBM analysis: smaller volume in the bilateral cerebellum tosil (A); larger adjacent white matter volume in the anterior cingulate cortex (ACC) and the midcingulate cortex (MCC)/posterior cingulate cortex (PCC) (B), as well as the bilateral putamen (C). For display purpose, images were thresholded at P<0.001, uncorrected.

In comparison with non-smokers, the chronic smokers displayed significantly smaller GM volume in cerebellum tosil (left, MNI [x = −34, y = −46, z = −44], peak z = 3.82; right, MNI [x = 26, y = −40, z = −46], peak z = 3.95, P_FWE_<0.05, small volume correction (svc)), as well as larger WM volume in the left ACC (MNI [x = −10, y = 44, z = 8], peak z = 3.63, P_FWE_<0.05, svc), the left midcingulate cortex (MCC)/posterior cingulate cortex (PCC) (MNI [x = −8, y = −42, z = 36], peak z = 3.78; MNI [x = −8, y = −38], z = 52 peak z = 3.60, P_FWE_<0.05, svc), and bilateral putamen (left, MNI [x = −18, y = 18, z = −2], peak z = 3.46; right, MNI [x = 22, y = 18, z = −4], peak z = 3.89, P_FWE_<0.05, svc). No regions were activated for other contrasts (smokers minus controls for grey matter or controls minus smokers for white matter). No significant correlation with other measurements (e.g. FTND) was found (Figure 1).

### Prediction accuracy

**Figure 2:**
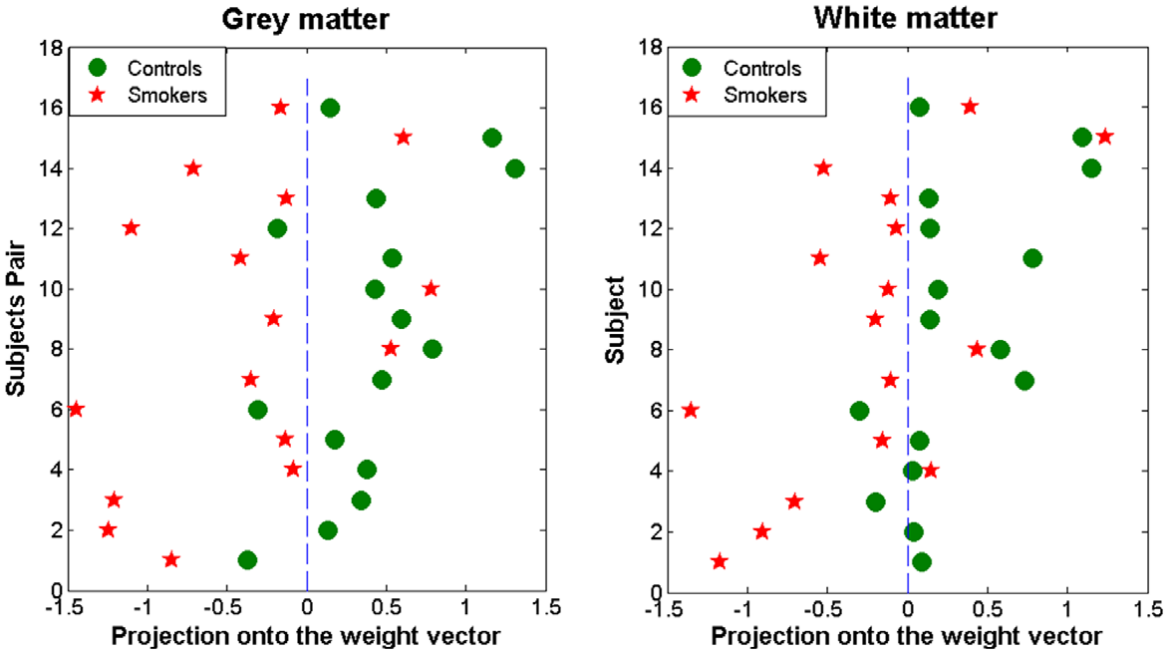
Classification accuracies for grey matter (left panel) and adjacent white matter (right panel) classifiers.

Fig. 2 summarizes the results of the classification between smokers and controls utilizing GM and WM images. The classification accuracy was 81.25% for using GM images. The sensitivity of the GM classification was 81.25%; i.e. if a subject was a smoker, the probability that this subject was correctly assigned to the smoker category was 0.81. The specificity of the GM classification was 81.25%, i.e. if a subject was a nonsmoker, the probability that this subject was correctly assigned to the nonsmoker category was 0.81. The classification p value resulting from the permutation test was very low, p<0.001, suggesting that GM images provide above chance classification accuracy. The similar classification accuracy was achieved for using WM images (accuracy = 81.25%, sensitivity  = 75.00%, specificity = 87.50%, classification p<0.001).

### Discrimination maps

**Figure 3:**
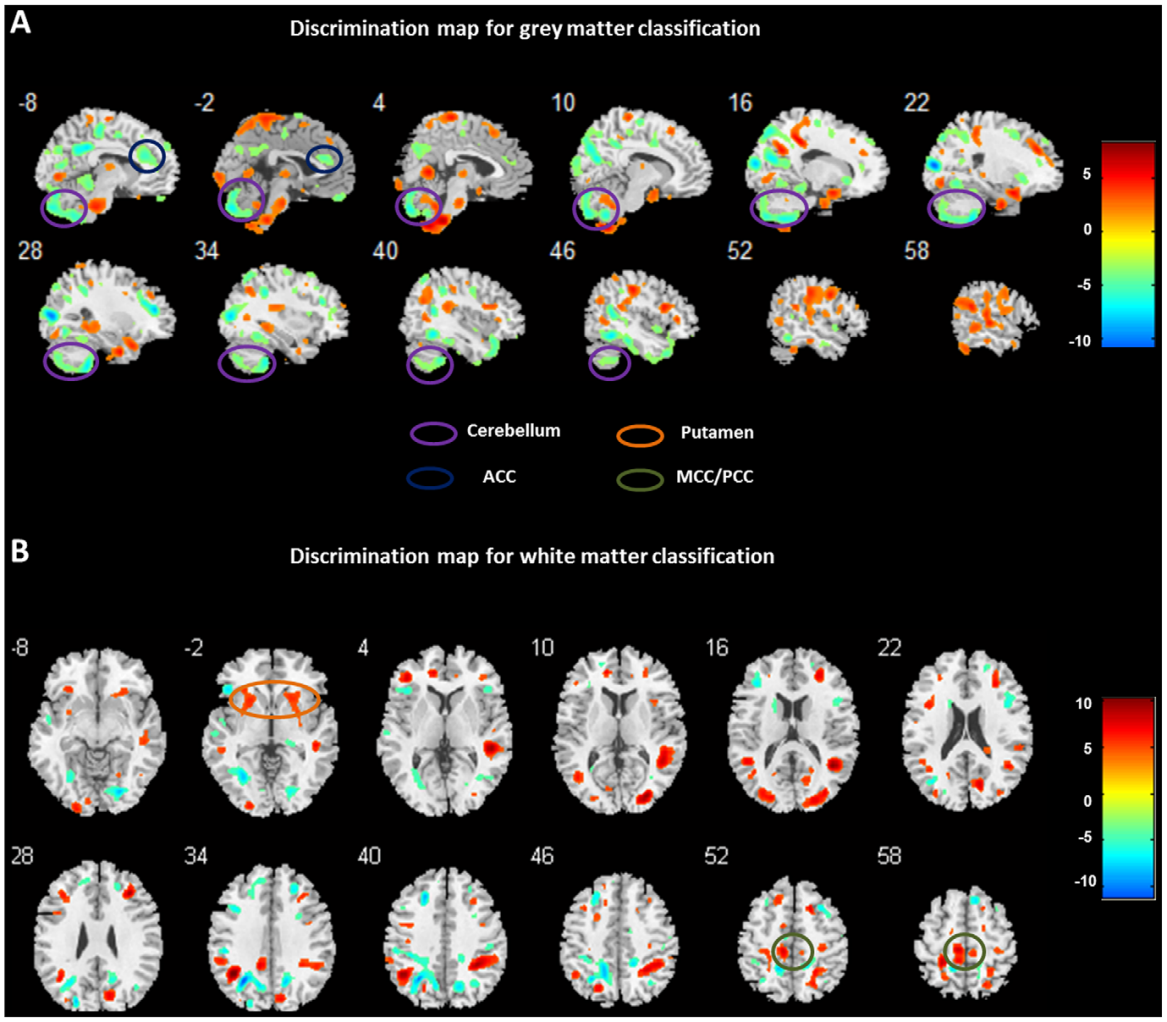
Discrimination map for grey (A) and white matter (B) classification. Red indicates higher values in the smokers than non-smokers, while blue indicates higher values for the non-smoker group than the smoker group. These regions were identified by setting the threshold to the top 30% of the weight vector scores. The x-coordinate for each sagittal slice and y-coordinate for each coronal slice in the standard Talairach space are given in millimetres.

Overall, the regions identified by SVM were similar to regions identified by VBM. The discrimination maps for GM classification showed smaller GM volumes in cerebellum, ACC, and other brain regions in smokers (see Table 1 and Figure 3). Moreover, multivariate analysis also found larger GM volumes in several regions in smokers, including parahippocampa gyrus, posterior cingulate cortex, and other areas. The discrimination maps for WM classification revealed larger WM volumes in ACC, MCC/PCC, putamen, as well as other regions including the superior temporal cortex, inferior parietal cortex, and middle occipital gyrus in smokers. In addition, smaller WM volumes were found in cerebellum, pons, and other brain regions in smokers.

**Table 1:** Most important gray and adjacent white matter regions discriminating between heavy smokers and controls.

Lobe	Brain Regions	W_i_	MNI Coordinates		
			X	Y	Z
	**Gray Matter**				
	Nonsmokers minus smokers				
Frontal lobe	Frontal subgyral	7.25	−38	12	24
	Superior Frontal Gyrus	8.35	28	42	16
	Precentral Gyrus	9.25	14	−28	72
	Anterior Cingulate	5.47	−14	46	6
Temporal lobe	Cortex	7.36	44	−50	−12
Parietal lobe	Fusiform Gyrus	7.81	−38	−52	34
	Supramarginal Gyrus	8.19	−10	−42	34
Occipital lobe	Precuneus	11.01	24	−88	12
Other	Middle Occipital Gyrus	8.38	−28	−40	−46
	Cerebellum Tonsil	7.40	28	−40	−50
	**Gray Matter**				
	Smokers minus nonsmokers				
Frontal lobe	Postcentral Gyrus	6	46	−24	42
Temporal lobe	Superior Temporal	5.73	60	−20	0
Parietal lobe	Gyrus	6.24	−22	−78	30
	Cuneus	6.52	−26	−65	−2
Occipital lobe	Lingual Gyrus	6.06	18	6	−20
Other	Parahippocampa Gyrus	6.04	−10	−32	−36
	Pons	6.39	2	−58	4
	Posterior Cingulate				
	cortex				
	**White Matter**				
	Nonsmokers minus smokers				
Frontal lobe	Medial Frontal Gyrus	6.10	−12	44	32
		7.03	24	36	34
Temporal Gyrus	Inferior Temporal	7.78	−40	−12	−34
Parietal lobe	Gyrus	11.03	−28	−60	34
	Precuneus	8.25	24	−80	−8
Occipital lobe	Lingual Gyrus	8.15	−28	−62	−4
Other	Middle Occipital Gyrus	6.86	−22	−56	−46
	Cerebellar Tonsil	9.99	−10	−32	−38
	Pons				
	**White Matter**				
	Smokers minus nonsmokers				
Frontal lobe	Anterior Cingulate	7.30	−12	44	6
	Cortex	7.45	−10	−42	−2
Temporal lobe	Midcingulate Cortex/	9.53	44	−50	16
Parietal Lobe	Superior Temporal	8.93	34	−42	40
Occipital lobe	Cortex	9.71	28	−86	12
Other	Inferior Parietal Cortex	5.62	22	18	52
	Middle Occipital Gyrus	5.72	−20	18	−4
	Putamen				

W_i_, weight of each cluster centroid *i*.

## Discussion

This is the first study focusing on both regional gray and white matter in long term, heavy smokers. Using a univariate VBM method, our study found that compared with controls, heavy smokers exhibited significantly smaller volumes in cerebellum, as well as significantly larger volumes in putamen, anterior and middle cingulate cortex. Using a multivariate patter classification method, we confirmed that these regions, together with other regions, distinguished smokers from non-smokers.

Consistent with two previous structural MRI studies [4], [7], we found significant GM reduction in cerebellum in heavy smokers. The cerebellum is rich in nicotinic cholinergic receptors [14]. Nicotine, the major biologically active substance that promotes smoking, binds to nicotinic cholinergic receptors and may exert its biological effects in cerebellar circuitry through these receptors [15], [16]. It is found that smokers showed an increase in cerebral blood flow in cerebellum after smoking a cigarette, suggesting that smoking influences cerebral activity [17]. The cerebellum is also implicated in other types of addiction. The cocaine-dependent group had lower gray matter volumes in bilateral cerebellum, which were negatively correlated with the duration of cocaine use [18]. Cerebellum was activated during cocaine craving [19]–[21], during recall of cocaine-use [22], and during stimulant expectancy [23]. Our findings, together with other studies, suggest that the cerebellum may be an important target for the chronic effects of smoking or drug addiction in general.

Out study provides the first evidence that adjacent white matter volumes in putamen and cingulate cortex were larger in smokers than in controls. The bilateral putamen is associated with craving and reward, and their hyperactivity in smokers when exposed to environmental cues that trigger craving has been documented by several studies [24], [25]. The putamen is known to be rich in dopamine, one of the key neurotransmitters playing a major role in addiction. In vivo nicotine and smoke exposures modulate subcellular organelle localization of dopamine D1 and D2 receptors in the caudate-putamen areas of adult rat brain [26]. The functional consequence of increased putamen WM volume in smokers may not be beneficial but indicate abnormal motivational functions. Reduced functional connectivity between putamen and cingulate cortex was found to be correlated with nicotine addiction severity [27]. A recent case study reported that a woman with a history of an addiction to cigarette smoking reported an immediate and complete disruption of her addiction to cigarette smoking following after posterior cingulate damage [28]. This observation suggests that the posterior cingulate cortex may also play an important role in the addiction to cigarette smoking. Together with the present work, these findings highlight an important role of putamen and cingulate cortex in nicotine addiction.

The GM and WM abnormalities in heavy smokers are further supported by the findings from multivariate pattern classification analysis (i.e. SVM). Moreover, the SVM analysis also revealed more brain regions that discriminated smokers and controls than those identified in traditional univariate analysis. Unlike conventional mass-univariate analysis (i.e., VBM), which considers each voxel as a spatially independent unit, SVM is a multivariate technique and considers inter-regional correlations. Individual regions may therefore display high discriminative power for several reasons, e.g. there is a large difference in volume between groups in that region, or this region is highly inter-correlated with other network components. Thus, discriminative networks should be interpreted as a spatially distributed pattern rather than making assumptions on their constituent parts. The voxel-by-voxel comparison approach on its own may lead to over-conservative findings. Employing SVM alongside VBM could greatly help to identify core structures implicated in nicotine addiction. For example, the GM volumes in ACC were found significantly smaller in smokers in multivariate analysis but that region was only found significant with a liberal threshold in VBM analysis (p<0.005, uncorrected). The findings that chronic smoking was associated with structural abnormality in widespread SVM clusters suggest smoking may influence a distributed network of brain regions rather than just certain brain regions. It is consistent with previous research showing that Nicotine replacement modulates large-scale brain network dynamics in the resting state [29].

Some limitations in our study are worth mentioning. First, the sample size in this study is quite small, which may result in Type 2 error. More work will, however, be required to investigate those relationships using a larger sample size. Second, we have to point out that the results in the present study must be interpreted with caution because it failed to survive the FDR or family-wise error (FWE) correction. Third, the designation of smoking status was based on self-report rather than confirmation with biological measures (e.g., breath carbon monoxide level). Some nonsmokers in our study may have had some history of smoking behavior in the past, which was not directly assessed in the present study. The impact of such a confound may not change the direction of the results since inclusion of former smokers in the nonsmoking group would have minimized rather than maximized differences in volumetrics between the two groups. However, the impacts of previous smoking and withdrawal on brain morphometry are still unknown and thus our results should be interpreted with caution. Fourth, we did not have detailed information on alcohol assumption. It is also possible that potential unrecorded group differences contributed to the findings. Fifth, we only examined male smokers and our results may not be generalized to female smoking population. Finally, the relative threshold 0.1 may not be strict enough to exclude the partial volume effect (when a pixel represents more than one kind of tissue type) and the smoothness may result in the inclusion of other brain areas. For example, it is possible the bilateral anterior limb of the internal capsule may also contribute to the observed volume enlargement in putamen rather than the putamen itself since the putamen has high grey matter/white matter ratio [30]. Although we did small volume correction using anatomical masks, such possibilities cannot be ruled out.

Despite these limitations, the present study had several strengths, including the use of a homogenous sample, and the examination of white matter and the use of multivariate pattern classification method. This study revealed abnormal structure in cerebellum, cingulate cortex, putamen, and other brain regions in heavy smokers. These findings enhance our current understanding of the neurobiology of chronic smoking.

## Materials and Methods

### Participants

Participants were recruited from the community through advertisements. Data were collected from 16 cigarette smokers and 16 matched healthy nonsmoking controls. All subjects were screened for psychiatric and nonpsychiatric medical disorders using the Structured Clinical Interview for the *Diagnostic and Statistical Manual of Mental Disorders*, 4th edition (DSM-IV; SCID). The cigarette use survey and Fagerström Test for Nicotine Dependence (FTND) were administered to each smoker by interview [31]. Prior to magnetic resonance imaging (MRI) scanning, urine drug screening was performed on all subjects to exclude substance abuse, with the exception of cigarettes. Additional inclusion criteria for smokers included men who met the DSM-IV criteria for current nicotine dependence (smokers) and smoked at least 15 cigarettes per day for at least 10 years. All nonsmokers in this sample never had a history of smoking. All subjects were right-handed and male, had no history of any neurological or psychiatric disorder, had no other drug dependence, and were not currently taking any medications. All subjects gave written informed consent. The study was approved by the Peking University Research Ethics Board.

### Image acquisition

All data were acquired on a Siemens 3-T Tim Trio Magnetic Resonance Imaging scanner (Siemens, Germany) by using a standard head-coil system. A high-resolution structural magnetization prepared rapid gradient echo scan (voxel size = 1.3×1.3×1 mm, repetition time = 2250 ms, echo time = 2.99 ms, inversion time = 900 ms, flip angle = 9°) was acquired in all participants.

### Image preprocessing

VBM analysis was performed in SPM5 (Welcome Trust Centre for Neuroimaging, London, UK), which enables automated spatial normalization, tissue classification, and intensity inhomogeneity correction to be combined within the segmentation step. Global GM/WM volumes were used as covariate to take into account the gross differences caused by spatial normalization. Default values for segmentation and normalization within SPM5 were used. Following normalization and segmentation into GM and WM, a modulation step was incorporated to take into account volume changes caused by spatial normalization which can cause certain brain regions to shrink or expand. This was done by multiplying the voxel values in the segmented images by the Jacobian determinants from the spatial normalization step. The segmented, normalized, and modulated images were smoothed using a Gaussian kernel of 8-mm full-width at half-maximum (FWHM).

#### Statistical analysis

An absolute threshold mask was set at 0.1 to ensure that the voxels included in the analysis had a higher probability of being WM or GM, respectively. Global WM and global GM volumes were calculated from segmentations in native space. Global WM volume was included as a covariate of no interest in the VBM analysis of WM volume differences, to account for any gross differences in total WM volume across participants [32]. Similarly, global GM volume was included as a covariate of no interest when changes in GM volume were being modelled. Age was also included as covariates of no interest in all models. We also performed a whole brain regression analysis to determine which regions correlated with clinical measures in the smoker group.

Small volume correction (svc) was used on a priori regions of interest including the putamen, anterior cingulate cortex, midcingulate cortex/posterior cingulate cortex, and cerebellum. There regions have been identified in previous studies in smokers 4–8. All ROIs were anatomical regions defined using the atlas in Pickatlas [33]. Activations in other regions were thresholded using a false discovery rate correction of P_FDR_<0.05 for the whole-brain volume with a minimum cluster extent of 50 contiguous voxels.

### Classification and support vector machine

After preprocessing, a linear support vector machine (SVM) as implemented in the PROBID software package (http://www.brainmap.co.uk/probid.htm) was used for image classification [11], [34]. Individual brain scans were treated as points located in a high dimensional space defined by the gray or white matter values in the preprocessed images. A linear decision boundary in this high dimensional space was defined by a “hyperplane” that separated the individual brain scans according to a class label (i.e. smokers vs. controls). If the input space is one dimension per voxel, the weight vector normal to the hyperplane will be the direction along which the images of the two groups differ most. Hence, it can be used to generate a map of the most discriminating regions (i.e., a discrimination map). In the GM/WM maps, the value in a voxel is correlated with the regional volume. Given two groups, patients and controls, with the labels +1 and −1, respectively, a positive value in the discrimination map (red scale) means relatively higher GM/WM matter volume in patients than in controls and a negative value (blue scale) means relatively higher GM/WM volume in controls than in patients. Because the classifier is multivariate by nature, the distribution of weights over all voxels can be interpreted as the spatial pattern by which the groups differ (i.e., the discriminating pattern).

To identify the set of voxels with the highest discriminative power, SVM recursive feature elimination was applied in order to remove as many non-informative features as possible while retaining features that carry discriminative information. A linear kernel matrix was used in order to reduce the risk of over-fitting the data and to allow direct extraction of the weight vector as an image (i.e. the SVM discrimination map). A “leave-one-out” cross-validation method was used which involved excluding a single subject from each group and training the classifier using the remaining subjects; the subject pair excluded were then used to test the ability of the classifier to reliably distinguish between categories (i.e. smokers vs. controls). This procedure was repeated for each subject pair in order to assess the overall accuracy of the SVM. Statistical significance of the overall classification accuracy was determined by permutation testing; this involved repeating the classification procedure 1000 times with a different random permutation of the training group labels and counting the number of permutations achieving higher sensitivity and specificity than the true labels. We repeated this procedure for gray and white matter images separately in order to assess the predictive power of each tissue type. Regions were identified by setting an arbitrary threshold of the top 30% of the weight vector scores [11], [13].
